# Perceived Cause, Environmental Factors, and Consequences of Falls in Adults with Cerebral Palsy: A Preliminary Mixed Methods Study

**DOI:** 10.1155/2015/196395

**Published:** 2015-02-23

**Authors:** Prue Morgan, Rachael McDonald, Jennifer McGinley

**Affiliations:** ^1^Department of Physiotherapy, School of Primary Health Care, Monash University, Frankston, VIC 3199, Australia; ^2^Department of Occupational Therapy, School of Primary Health Care, Monash University, Frankston, VIC 3199, Australia; ^3^Department of Physiotherapy, School of Health Sciences, The University of Melbourne, Parkville, VIC 3010, Australia

## Abstract

*Objective*. Describe perceived cause, environmental influences, and consequences of falls or near-falls in ambulant adults with cerebral palsy (CP). *Methods*. Adults with CP completed postal surveys and follow-up semistructured interviews. Surveys sought information on demographic data, self-nominated Gross Motor Function Classification Score (GMFCS-E&R), falls, and near-falls. Interviews gathered additional information on falls experiences, near-falls, and physical and psychosocial consequences. *Results*. Thirty-four adults with CP participated. Thirty-three participants reported at least one fall in the previous year. Twenty-six participants reported near-falls. Most commonly, falls occurred indoors, at home, and whilst engaged in nonhazardous ambulation. Adults with CP experienced adverse falls consequences, lower limb injuries predominant (37%), and descriptions of fear, embarrassment, powerlessness, and isolation. *Discussion*. Adults with CP may experience injurious falls. Further investigation into the impact of falls on health-related quality of life and effective remediation strategies is warranted to provide comprehensive falls prevention programs for this population.

## 1. Introduction

Cerebral palsy (CP) is the commonest cause of neurological disability in childhood [1]. With now near-normal life expectancy for many adults with CP anticipated, it represents a growing population in adults living with disability [2]. However, as they age, individuals with developmental disabilities such as CP often experience health issues associated with accelerated ageing such as pain, fatigue, under- and overnutrition, premature sarcopenia, and osteoporosis [3]. This occurs at a younger age than that of their nondisabled peers and at higher rates than the general population [4]. Recent publications have begun to explore premature mobility decline, balance dysfunction, and falls in adults with CP and confirmed the increased prevalence in young and middle aged adults [5–7].

The issue of falls in adults with CP is of concern, as persistent falls may occur earlier than the typical “older adult” focus in nondisabled populations. One or more falls in the previous two years has been reported by 18% of a normative group aged 20–45 years and 21% of a normative group 46–65 years [8]. The relationship between age and falls in normative populations is well established [8] with an escalation of falls frequency related to increased age. However such a relationship has not been described in adults with CP. At present, the literature in the area of adults with CP and falls is sparse, but what is available suggests reason for concern. One study [9] showed that 40% of adults with CP fell monthly, and 75% fell bimonthly in a sample with a mean age of 44 years. In a cohort of young adults with spastic diplegia, 3/16 participants were falling more than 50 times over a 12-month period [7], and Morgan and McGinley reported that 68% of a small cohort of adults (mean age 41 years) experienced one or more falls in six months [10]. The implications of such falls in this population are not well described.

Precipitators, patterns of injury, and psychosocial outcomes have been well published arising from falls in older adults and those with acquired neurological disorders [8, 11–13]. For example, adults with Parkinson's disease typically fall within their own home [14], and estimates of soft tissue injury rate following a fall in those following strokes range from 24 to 48% [13]. Older adults who fall may report feelings of powerlessness, fear, loss of control, and anxiety as a result [15]. Falls in older adults have been associated with injury, loss of independence, and increased health service usage [16], with hospitalisation, rehabilitation, and associated carer costs. As a result of this condition-specific knowledge, effective falls prevention programs have been targeted and implemented for older adults and those with acquired neurological dysfunction [13, 17–19].

Emerging evidence suggests that moderate or serious injuries do result from falls in adults with CP [5] and people experience an elevated level of fear of falling as a result [5, 7]. The nature and impact of falls on adults with CP with longstanding mobility dysfunction may differ to those who have more recently acquired mobility and balance problems such as stroke or Parkinson's disease. The causation, specific information regarding physical injuries incurred, and a deeper understanding of the psychosocial consequences of falls in adults with CP are currently not known. Falls prevention programs that are tailored for adults with CP currently do not exist. Greater evidence regarding the lived experience of falls as described by adults with CP will enable the development of targeted falls prevention programs to address both the physical and psychosocial consequences of falls in this population.

The aim of this preliminary mixed methods study was to explore the lived falls experience of adults with CP. Specifically, this included an exploration of the perceived cause, environmental influences, and consequences of falls and near-falls in ambulant adults with CP. A further aim was to explore the relationship with falls and age in adults with CP.

## 2. Methods

Ethical approval was gained from Monash University (MUHREC, LR, 2011001612). The study comprised two elements, an initial survey and a follow-up semistructured interview.

### 2.1. Study Sample

Flyers advertising the study were placed at physiotherapy clinics, health facilities, and community agencies. Interested participants were invited to contact the researcher whereby further information about the study was provided and a short interview clarified inclusion criterion. Inclusion criteria for the study were adults with CP (age 18–65 years), with Gross Motor Function Classification System–Expanded & Revised (GMFCS-E&R) levels I–III [20]. Participants were excluded if English language comprehension or cognitive impairment (abbreviated mental test score <7/10) [21] precluded the ability to follow instructions to enable survey completion. Following survey completion, participants were invited to self-select for a follow-up semistructured interview by completing a permission slip at the conclusion of the survey indicating their willingness to be contacted by the researcher.

### 2.2. Measurement Tools and Procedures

A survey was developed and piloted whereby consumer feedback was sought regarding the language, descriptors, and layout. The survey was then modified to improve clarity. The survey provided initial lay-definitions of both a fall (“an unexpected event in which you come to rest on the ground, floor or lower level”) [22] and near-fall (“a stumble or loss of balance that would result in a fall if you could not recover in time”) [23]. The survey sought detailed information about the person and their experience with falls and took approximately 30 minutes to complete. Additional data regarding health status and wellbeing were captured within the survey but are reported elsewhere [24]. The survey was then mailed to participants with a reply paid envelope or distributed electronically via email if requested.

Participant demographic characteristics were recorded in the areas of age, gender, and CP subtype (if known) [25]. Current level of mobility was measured using the GMFCS-E&R [20]. The GMFCS-E&R is a scale that classifies gross motor function into five levels, with Level I describing the highest level of function. Ambulant category descriptors were as follows: Level I walks without limitations; Level II walks with limitations; Level III walks using a hand held mobility device [26]. Levels IV and V refer to nonambulant descriptors. As falls in a nonambulant population are likely to have different characteristics, only ambulant participants (Levels I–III) were included in this study. Self-nomination of current GMFCS-E&R level has been shown to have excellent agreement with professional ratings [27].

Participants recorded their estimated number of falls in the previous 12 months. A series of linked survey questions probed the circumstances and consequences of the individual's most recent fall, either by selection of multiple choice items or by open questions (free text options). Thus participants reported the likely fall cause (trip, slip, lost balance, dizzy, knees gave way, faint, medication/alcohol, or other) [28], location (inside at home, outside at home, or in the community), precipitating activity immediately prior to the fall, any obstacles contributing to the fall, time of day (am or pm), injuries sustained, and medical attention sought (none, local doctor, emergency department, or other). Participants also rated their typical frequency of near-falls as daily, weekly, monthly, less than monthly, or no near-falls.

A semistructured interview guide was developed to explore the following topics: experiences of falls/near-falls; perceptions of change in falls and/or near-falls type and frequency since adolescence; physical health consequences of falls/near-falls; and psychosocial health consequences of falls/near-falls. To clarify responses and identify examples, the interviewer rephrased the questions and asked follow-up questions if required [29]. The interview also explored participants' experiences of health service access and utilisation. This latter data has been reported elsewhere [30]. A pilot interview was conducted to refine language and trial the interview guide, with these data (Participant 1) included in the final analysis.

A mutually convenient interview appointment was made for participants who chose to be interviewed. Interviews were conducted face to face at the person's home, with the option of a telephone interview if geographical distance necessitated. A consent form was forwarded seeking signed permission for participation, audio-taping of the interview, and subsequent transcription. The interview lasted up to an hour, with rest breaks available if requested. Two research assistants conducted the interviews. The interviewers, a final year health science student and a physiotherapist, had basic knowledge in disability care and had no treatment connections with the participants. The interviewers completed a mock interview on another member of the research team to refine their interview technique prior to commencing data collection.

### 2.3. Data Analysis

Quantitative data were entered into the SPSS statistical software version 19.0 (SPSS Inc, Chicago, Illinois). Descriptive statistics were used to report age, gender, CP subtype, GMFCS-E&R level, reported falls, and reported near-falls. Based on the methodology used by O'Loughlin and colleagues [31], participants were characterised as repeat fallers (fell > 2 times in the previous year) or infrequent or nonfallers (fell ≤ 2 times in the previous year). The relationship between age and falls frequency was tested with scatter plots and Spearman* rho* (*r*_*s*_) correlation coefficient, with significance set at *P* < 0.05.

Text responses regarding the most recent fall were entered into a spread sheet. PM and a research assistant independently matched responses against categories previously defined by Hill and Stinson [32] to describe precipitating activity, obstacles contributing, and fall location. Injury was subsequently categorised as injury type (none, bruise, cut, sprain, bump, and fracture) adapted from Talbot's coding [8] and attributed to body part. If discrepancies in categorisation of response occurred, consensus was achieved by the use of a third independent researcher. Free text response categories were reported using frequencies.

Interview data were transcribed to written text, checked by a second researcher, and then imported into Nvivo software (QSR International P/L). Researchers used a phenomenological approach to uncover the meaning of an individual's experience of a specified phenomenon (a fall) through focusing on a concrete experiential account grounded in everyday life [33]. Analysis of data was conducted with provisional exploration and theoretical sampling of data performed after repeated reading of the text to identify significant statements and develop provisional themes [33]. Coding of all interview transcripts into themes was undertaken independently by PM and a research assistant. Different interpretations regarding thematic identification and the generation of textural descriptors [33] were compared and reviewed against the initial study aims. Following this, any discrepancies in key themes and descriptors were resolved by discussion. Data were concurrently reviewed after provisional analysis of every transcript in an iterative approach.

Data from the interviews were nested within the qualitative analysis in order to produce greater insight and give deeper context to the quantitative results than would be gained by a single method. Furthermore, the qualitative data enabled the gathering of the specific language and voices about experiences with falls as described by this population [34].

## 3. Results

The mean age of the 34 adults with CP who participated in the study was 44.2 years (sd 8.6, range 26–65). Nineteen participants were female, with ten each of spastic bilateral and unilateral CP subtypes. There were four each of the subtypes of athetoid and mixed (athetoid/spastic) with remainder unknown or unstated. Five participants rated their gross mobility as GMFCS-E&R Level I, 14 as GMFCS-E&R Level II, and 15 as GMFCS-E&R Level III. The participant group is reported elsewhere [24].

Thirty-three of 34 participants reported at least one fall in the past 12 months, ranging from no falls to an estimation of 200 falls, that is, around 4 falls per week (median 5, IQR 9.0). Further details regarding the falls profile of the participants are provided in Table 1. In addition to the self-reported falls, near-falls also occurred frequently in over 75% (26/34) of participants. Age was not found to be associated with falls frequency (*r*_*s*_ = −0.261, *P* = 0.136).

**Figure 1:**
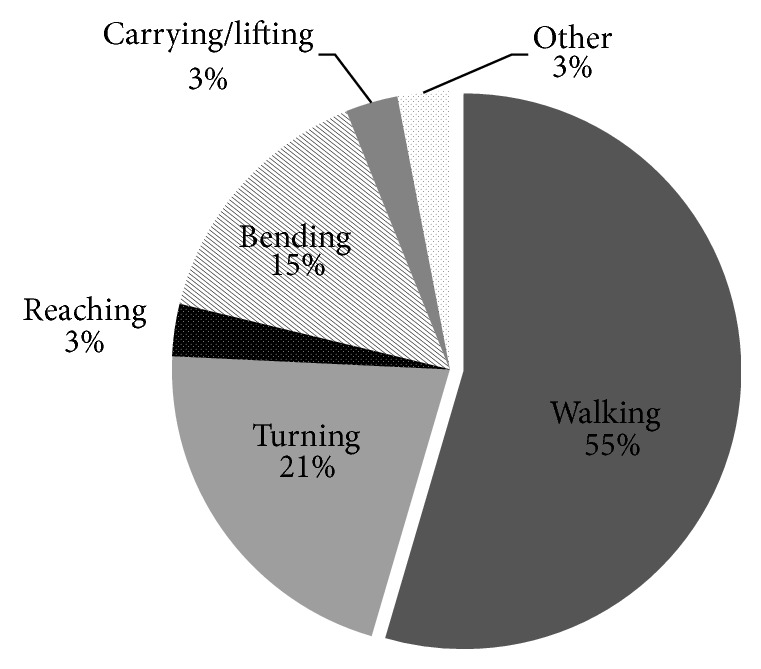
Distribution of activities undertaken prior to most recent fall.

**Figure 2:**
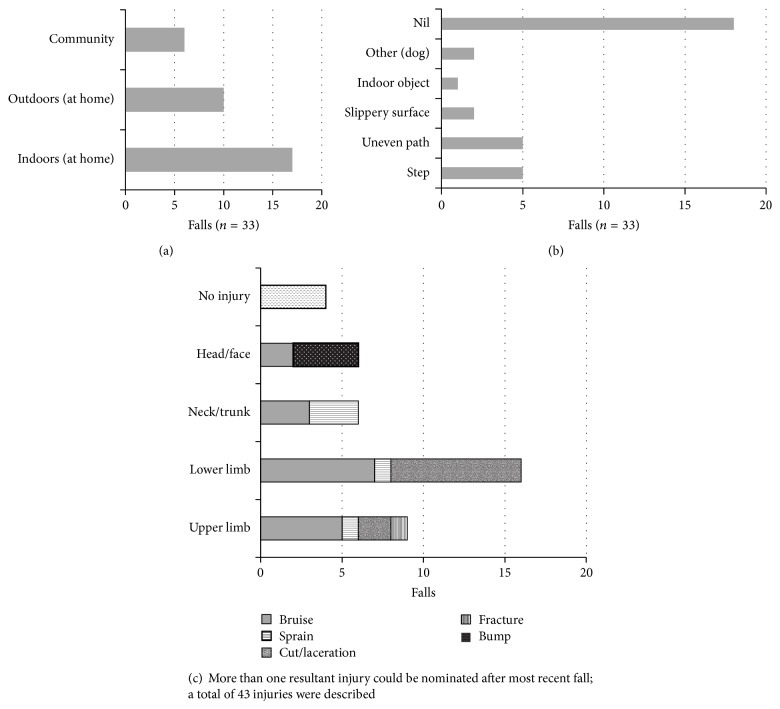
Location of most recent fall (a), obstacle involved in most recent fall (b), and consequence of most recent fall (c).

Six participants were classified as infrequent/nonfallers (fell ≤ 2 times) and 28 participants were classified as frequent fallers (fell > 2 times). Frequent falls were experienced in individuals across all levels of mobility ability, with 5/5 frequent fallers at GMFCS-E&R Level I, 11/14 at GMFCS-E&R Level II, and 12/15 at GMFCS-E&R Level III. The activities being undertaken at the time of the most recent fall (*n* = 33) (Figure 1), location of fall, contributing obstacles, and falls consequence were explored in more detail (Figure 2). Two injuries to the face required general practitioner (GP) follow-up for sutures, and one participant sought physiotherapy treatment for a back sprain. Two more serious injuries resulting in emergency department (ED) admissions occurred whilst out in the community (fracture, severe bruising to hip/back) (Table 1).

Six participants (mean age 45.8 years, range 35–52 years), five female, were self-selected from the original cohort for the follow-up interview. Five interviews were performed face to face, with one being completed via telephone. Three participants were at GMFCS-E&R Level II, and three at GMFCS-E&R Level III. Five of the six interview participants lived with family members or defacto, and three were currently employed (two full time, one part time).

A range of experiences regarding perceptions of falls and near-falls and the physical and psychosocial health consequences of these were described throughout the six interviews. Key themes that emerged were experiences of falls and near-falls and the impact of falls on physical and psychosocial health. Key themes and qualifiers are summarised in Table 2. The themes are illustrated by quotations where “…” indicate a pause and an explanation to a quotation is presented in brackets. The number in brackets after a quotation refers to a specific participant.

### 3.1. Falls and Near-Falls Experiences: “Getting on with Life”

By middle age (40 years onwards), most adults have established a routine in their daily life involving repetition of activities, employment, leisure, and relationships. Most people accept that part of living or “getting on with life” is dealing with minor obstacles or challenges along the way through either resolving that challenge, eliminating that obstacle, or developing tolerance or acceptance of the renewed situation [35]. All interview participants had experienced multiple falls as an adult. Participants described concepts of inevitability, tolerance, and acceptance of their regular falls or near-falls on their daily life. For one, the falls were long standing, commonplace, and taken relatively lightly:

*“falling over is just a part of life, I do not enjoy it but there's not much I can do about it” (Participant 5) *

and

*“I can fall over in the kitchen and nobody sees me, I do not give a s…, I do not even remember ok? I just pull myself up and get on with my life” (Participant 5).*

Other participants echoed this concept of tolerance of falls that had been experienced over an extended period of adulthood:

*“I had a really big fall just a couple of days ago…but you just have to get up and keep going” (Participant 6)*

or

*“falls being second nature, you learn to get on with them” (Participant 2).*

This implied that the falls experienced by adults with CP were not troublesome nor something to be overly concerned about, but a normal inoffensive part of their day that they had learned to live with as they “got on with their life.”

### 3.2. Falls and Near-Falls Experiences: “Increasing Risk Awareness”

The experience of falling throughout adolescence and young adulthood was becoming more problematic and more intrusive in daily life for some interviewees as the impact of the falls increased. A greater awareness of the potential to fall and the reality of a fall was now more dominant, requiring attention and focus in a range of environments:

*“I was falling a lot - up the stairs, in the hallway, walking along the street, uneven surfaces, flat surfaces, it just did not matter…” (Participant 3). *

This risk awareness was not necessarily related to falls frequency, with concern expressed by all participants regarding the potential for falls and the adverse impact of falls. For those who were not frequent fallers, managing the increasing falls risk had become a more prevalent aspect and an important focus of their daily life:

*“Even though I do not fall that often I'm constantly aware of it” (Participant 4).*

In addition to the fall itself, an increased awareness of the need to manage the sequelae was also becoming a necessity. For those who have valued independence in mobility, a fall could potentiate an inability to get up off the floor resulting in increased dependency. This added to the impact of a fall and required sustained attention to reduce the subsequent risk:

*“I've always fallen over, but I was able to get up, and just dust myself, and get on with life. But now, if I fall over, I cannot get up. So I'm sort of like a beached whale” (Participant 1).*

### 3.3. Impact of Falls on Physical Health (Injuries, Severity)

The impact of falls on physical health was a focus of many participants' interviews. For any adult sustaining an insult to their physical health, there is the immediate pain or “crisis,” followed by a period of recovery that can be relatively brief or long term. Adults with CP reported increasing concern with the adverse physical consequences of a fall, with descriptions of musculoskeletal damage to arms, hands, head, and spine and resultant symptoms of stress. They reported a range of short and longer term physical injuries as a result of falls that had minor or more serious implications, although these were typically reported in a pragmatic manner:

*“I did some pretty awful damage to an elbow, I dislocated a finger, I ended up having a CT scan because I fell down the stairs and landed on a wall headfirst” (Participant 3),*

and

*“I'm finding out that when I fall over, I cannot get up, and it causes me to get…have so much pain in my lower back, and I've broken it, so every fall I have, whether it's just…even if it's a near miss, where I've jolted myself, I have to take Valium” (Participant 1).*

One participant described the serious consequence of a minor physical injury from a fall:

*“I broke a tiny bone in my hand from falling and it meant that my hand was out of action for 6 weeks. I had to go to hospital, had to be in plaster. How do you push a wheelchair with one arm in plaster? It was just an absolute nightmare” (Participant 4).*

### 3.4. Impact of Falls and Near-Falls on Psychosocial Health (Fear, Embarrassment, Powerlessness, and Isolation)

In contrast to earlier expressions of tolerance and acceptance, psychosocial subthemes of fear, embarrassment, powerlessness and isolation were identified. In alignment with reports of fear of falling in older adults, most participants associated the falls and near-falls with negative emotions related to fear or increasing concern and described the subsequent adverse impact on their psychosocial health:

*“scared, worried, concerned about the future” (Participant 4); “I constantly have near misses, every day. And they do scare me quite a bit” (Participant 1). *

Participants described incongruence between their perceptions of appropriate adult and childhood motor behaviour, with “falls” being associated with more immature activity, a feature that should not persist into adulthood. Several participants described the embarrassment, humiliation, and negative impact on their self-esteem of continued or increased falls throughout adulthood:

*“You do that (fall) in a public place and everyone is like ‘adults do not fall over, little kids fall over…', so it's very embarrassing. It's something that I should get over but I haven't” (Participant 5) *

or

*“ I'm getting too old to fall, I'm 42, I cannot get away with it anymore. When you're a kid you can get away with it and everyone thinks you're being an idiot but I just cannot tolerate it anymore” (Participant 3).*

For one participant, repeated falls were particularly problematic, resulting in disorientation and concerns of negative perceptions of self by others. The humiliation that arose from the public fall was particularly difficult to endure:

*“One time I fell on the road and I couldn't get up and then I couldn't orientate myself and I'd fall again, I must have looked like a drunk” (Participant 3). *

Adults typically value control over their health. Some participants acknowledged adverse mood, depressive symptoms, or feelings of “loss” associated with their experience of falls and subsequent repeated injuries. A sense of powerlessness arose associated with the unpredictability but inevitability of the ongoing and escalating falls behaviour:

* “I've always valued my independence, and that's the thing that I miss most” (Participant 1)*

and

*“I'm not chronically depressed about it or anything but it's sad to know that I cannot really fix anything (the falls) so I just have to go day by day and do the best I can” (Participant 4).*

The falls, near-falls, declining balance, elevated falls risk, and associated health complications added to a strain on some people's capacity to maintain social contacts with a consequent developing sense of isolation:

* “now that I do not have that balance, I cannot negotiate the steps, which has really curtailed my social life” (Participant 1).*

## 4. Discussion

This study reported falls frequency, falls precipitators, and consequences (physical and psychosocial) and the relationship of falls to age for a group of ambulant adults with CP. There is a dearth of information in this area regarding the prevalence and consequences of falls in this group in order to guide both adults with CP and health professionals in best practice in falls prevention and management. Furthermore, there has been limited emphasis by both health care providers and health care seekers, on falls in those growing older with CP who may have experienced a “life time” of falls and near-falls.

Almost all survey respondents (33/34) reported one or more fall in the previous 12 months. This is an alarming number, exceeding that previously identified [7, 10] and likely reflects selection bias in this population. Nevertheless, for this group of individuals, falls with resulting injury are of concern. It may be that these relatively young adults are not adapting their activities to accommodate decline in mobility and balance function associated with premature ageing. Interestingly, the interview participants in this study appeared not to restrict activity as a result of falls, which would typically occur in older adults after fall or in those with acquired neurological dysfunction [12, 36]. It may be that ambulant adults with CP have experienced falls or near-falls over a long period of time and hence continue to pursue their activities in spite of the elevated falls risk. Alternatively, it may be that participants were already engaged in lower physical activity levels than age matched peers [37, 38], suggesting that scope for further reduction in activity level, which would reduce the risk of falls, may be limited.

In normative populations, falls frequency is typically low in those under 65 years [8]. This study failed to show a significant relationship between age and falls frequency in this population. However, qualitative data emphasizes the ongoing injurious falls behaviour in many from early adulthood onwards, with no evidence of a plateau in falls upon reaching musculoskeletal maturity. It was considered important to interpret the falls reports and injury descriptions from this study in the context of a person ageing with a disability, that is, in the context of a person who may already have limited or altered mobility. For example, whereas an upper limb injury may have minor consequences in an able bodied person, in an adult with CP it can make managing mobility aids problematic, decrease productivity, or necessitate additional personal care. Additional falls risk factors other than age should be considered when identifying and addressing falls risk in this population.

Serious injury reports by this cohort are slightly higher than that published after falls in older adults (5–10%) [39] and previously reported in ambulant adults with CP [5]. This adds to the growing body of evidence regarding falls in this population with unknown likely financial impact on health services and carers. Anecdotally, there is very limited emphasis on falls prevention education provided to adults with CP.

Ambulation has been cited as the predominant activity undertaken prior to a fall in normative studies, in all gender and age groups [8]. In the falls reports of adults with CP in this cohort, the majority were engaged in ambulatory nonhazardous tasks at the time of the fall. In normative data [8], neurological populations [32], and the adults with CP in this study, the environmental factor contributing to the highest percentage of falls was uneven surfaces/steps. This adds to the impetus to ensure safe surface access throughout private and community areas for those ageing with disability. A multidisciplinary review of care needs and home adaptations for older adults has been effective in addressing falls and function [40] and should be explored for adults ageing with CP.

To our knowledge, there have been no other qualitative studies describing psychosocial consequences of falls and near-falls experienced by adults with CP. It was apparent from the interviews in this study that although falls had been a frequent occurrence throughout adolescence and young adulthood, with many acquiring “expert faller” status [7], the increasing frequency resulted in a range of negative emotions such as fear, embarrassment, intolerance, and powerlessness. An increased fear of falling (as measured with the FES-I) has previously been described in ambulant adults with CP [5, 7]. Both fear and embarrassment have also been reported by Yardley and colleagues in their study of the negative consequences of falling in older adults, an expectation of “social embarrassment and indignity and consequent damage to personal confidence and identity” were reported [41]. Reports of powerlessness have also been described by older adults experiencing falls [15] and are associated with perceptions of the unpredictability and lack of control over falls. Fear, embarrassment, and perceptions of the “inevitability” of a fall impact on the ability of adults with CP to participate fully in all aspects of life and were identified in this study. These issues warrant further investigation in this group.

Adults with CP may have lived their life managing mobility and balance difficulties and hence may be quite used to experiencing falls, finding them a not unexpected disruption. Interestingly, there was tension in the qualitative data between the two themes of “getting on with life” and adverse psychosocial effects (fear, embarrassment, powerlessness, and isolation). For all participants, both themes were alluded to within the same interview, although often the “getting on with life” (minimisation of the falls impact) was the first theme to be raised. It may be that as familiarity with the interviewer developed over the session participants became more willing to expose their vulnerabilities. This study suggests that even those who have a long history of falls may find the ongoing falls experience or escalating falls experience intolerable in adulthood, with adverse psychosocial consequences. This phenomenon has not previously been described. Less than half of a large cohort of adults with CP surveyed by Jahnsen and colleagues [42] reported that they had learned how to take personal responsibility for their own health as adolescents/young adults. Heller and Sorensen [4] recently summarised the outcomes of 31 different health promotion programs for adults with intellectual and developmental disabilities, only one mentioned falls prevention as a target of the intervention [43]. In older adults and those with acquired neurological disability, strategies such as Tai Chi and multidisciplinary “Falls and Balance” programs have reduced falls frequency in vulnerable populations [13, 17–19], but the efficacy of these programs on falls in adults with CP is yet to be established. A recent publication has suggested that balance decline in ambulant adults with CP may be addressed by a small group outpatient program of individualised exercises [44]. Its impact on reducing falls frequency is unknown. The ability to quantify risk and provide evidence-based falls prevention education including tailored psychosocial support to adults ageing with CP is a desirable outcome of further research.

Six community falls were reported by this cohort, of which two required ED attention. There is increasing public interest in accessibility for people with disabilities. For example, both US and Australian standards [45, 46] for building and construction provide minimum requirements for access to most public buildings, fit out of public bathrooms and toilets, and guide housing design. Further work regarding access to the community is required with this and similar groups to explore issues that may be contributing to falls within the community environment, which occurred in this study. Individually, review of local environments by health professionals may be warranted. Application of universal design principles should be advocated [47].

The main limitation of this study was a bias in selection. A sample of convenience was sought through advertisements at clinical and community facilities, resulting in bias toward those who may already identify concerns regarding falls. It is also possible that recall bias influenced both underreporting and overreporting of falls details. However, we would argue that subjective experiences, such as falls are best explored by collecting data directly from those who experience them, rather than third party reports, although there is a risk that survey questions may be misunderstood or interpreted differently by the participant [42]. In addition, only a small number of interviewees were recruited, with a bias towards women. Despite these limitations, the additional descriptive information generated from the qualitative data in this study provided additional confidence in triangulation of data and subsequent outcomes. Finally, the findings from this study can only be generalised to cognitively able adults with CP living in private homes.

## 5. Conclusion

In conclusion, this study has illustrated that adults with CP may experience falls and incur both adverse physical and a range of psychosocial consequences. Falls may occur due to balance dysfunction, occur indoors and outdoors, and occur predominantly at home and whilst people are undertaking nonhazardous activities. Further investigation into the impact of falls on participation, health related quality of life, and effective remediation strategies is warranted in order to develop appropriate falls prevention strategies for this population.

## Figures and Tables

**Table 1:** Falls data including falls and near-falls number, cause, time of fall, and injury sustained.

Falls profile	
Falls in past 12 months (*n* = 34)	0 falls: 1 1-2 falls: 5 3–6 falls: 17 7–19 falls: 4 20–60 falls: 3 >60 falls: 4

Near-falls (*n* = 34)	8 none 9 daily 12 weekly 0 monthly 5 less frequently than monthly

Cause of most recent fall (*n* = 33)^*^	Balance loss: 28 Trip: 6 Slip: 6 Dizzy: 1

Time of most recent fall (*n* = 33)	12 morning 21 afternoon/evening

Injury sustained from most recent fall (*n* = 33)	6 none 22 minor, no medical attention 3 minor, medical attention (GP or physio) 2 severe (ED admission)

^*^More than one contributor could be selected; GP: general practitioner; ED: emergency department.

**Table 2:** Key themes arising from interviews.

Key theme	Qualifier
Falls and near-falls experiences	Getting on with life Increasing risk awareness

Impact of falls and near falls	Physical health: injuries and severity Psychosocial health: fear, embarrassment, powerlessness, and isolation
